# Cytosolic and Nucleosolic Calcium-Regulated Long Non-Coding RNAs and Their Target Protein-Coding Genes in Response to Hyperosmolarity and Salt Stresses in *Arabidopsis thaliana*

**DOI:** 10.3390/ijms26052086

**Published:** 2025-02-27

**Authors:** Doudou Wang, Kaifeng Zheng, Wenfen Long, Liang Zhao, Wanjie Li, Xiuhua Xue, Shengcheng Han

**Affiliations:** 1 Beijing Key Laboratory of Gene Resources and Molecular Development, College of Life Sciences, Beijing Normal University, Beijing 100875, China; wangdoudou9408@163.com (D.W.); kaifeng_zheng@mail.bnu.edu.cn (K.Z.); longwf931@163.com (W.L.); zhaoliang20181012@163.com (L.Z.); lwj@bnu.edu.cn (W.L.); 2 Academy of Plateau Science and Sustainability of the People’s Government of Qinghai Province & Beijing Normal University, Qinghai Normal University, Xining 810008, China

**Keywords:** cytosolic calcium signal, nucleosolic calcium signal, long non-coding RNA, salt stress, hyperosmolarity stress

## Abstract

Long non-coding RNAs (lncRNAs) are involved in plant biotic and abiotic stress responses, in which Ca^2+^ also plays a significant role. There is diversity in the regulation of different gene expressions by cytosolic Ca^2+^ ([Ca^2+^]_cyt_) and nucleosolic Ca^2+^ ([Ca^2+^]_nuc_). However, no studies have yet explored the interrelationship between lncRNAs and calcium signaling, nor how calcium signaling regulates the expression of lncRNAs. Here, we use transgenic materials *PV-NES* and *NLS-PV*, which simulate [Ca^2+^]_cyt_- and [Ca^2+^]_nuc_-deficient mutants, respectively, and wild type (WT) materials in response to hyperosmolarity (250 mM sorbitol) or salt stresses (125 mM NaCl) at different time points to obtain RNA-seq data, respectively. Then, we proceed with the screening of lncRNAs, adding 688 new lncRNAs to the known Arabidopsis lncRNA database. Subsequently, through the analysis of differentially expressed lncRNA genes, it was found that cytosolic or nucleosolic calcium signals have distinct regulatory effects on differentially expressed lncRNAs (DElncRNAs) and differentially expressed protein-coding genes (DEPCGs) treated with high-concentration NaCl and sorbitol at different times. Furthermore, through weighted correlation network analysis (WGCNA), it is discovered that under hyperosmolarity and salt stresses, lncRNA-associated PCGs are related to the cell wall structure, the plasma membrane component, and osmotic substances through *trans*-regulation. In addition, by screening for *cis*-regulatory target PCGs of Ca^2+^-regulated lncRNAs related to osmotic stress, we obtain a series of lncRNA-PCG pairs related to water transport, cell wall components, and lateral root formation. Therefore, we expand the existing *Arabidopsis* lncRNA database and obtain a series of lncRNAs and PCGs regulated by [Ca^2+^]_cyt_ or [Ca^2+^]_nuc_ in response to salt and hyperosmolarity stress, providing a new perspective for subsequent research on lncRNAs. We also explore the *trans-* and *cis*-regulated target PCGs of lncRNAs regulated by calcium signaling, providing new insights for further studying salt stress and osmotic stress.

## 1. Introduction

Long non-coding RNAs (lncRNAs) are a class of RNAs longer than 200 nucleotides, which are characterized by low coding potential or no coding ability and typically exhibit cell or tissue specificity [1]. Based on their origin relative to protein-coding genes (PCGs), lncRNAs are currently classified into four categories: long intergenic non-coding RNAs (lincRNAs) derived from regions between genes, intronic lncRNAs (incRNAs) derived from intronic regions of PCGs, natural antisense lncRNAs (NATs) derived from the antisense strand of PCGs, and sense lncRNAs derived from exons of PCGs [2]. Recent research has shown that lncRNAs primarily regulate the expression of neighboring genes in a *cis*-regulatory manner and that of distant genes in a *trans*-regulatory manner [3]. The regulatory mechanisms of lncRNAs on target genes are specifically manifested as chromatin remodeling, transcriptional regulation, R-loop formation, and encoding precursors of miRNAs or siRNAs, competing with miRNAs for binding and alternative splicing [4]. With the rapid advancement of RNA sequencing technology and the deepening understanding of lncRNAs, a substantial number of novel lncRNAs have been identified in various plant species, including Arabidopsis [5], rice [6], barley [7], and tomato [8], among other plants. Accumulating evidence suggests that lncRNAs play crucial roles in regulating plant growth and development, as well as in mediating responses to both biotic and abiotic stresses [9].

As the global average temperature rises, the rate of surface water evaporation accelerates, and water scarcity-induced droughts as well as soil salinization become increasingly severe; plants, especially crops, are facing significant challenges [10]. Plant roots, being in direct contact with the surrounding environment, play a crucial role in sensing salt stress and osmotic stress caused by drought, as well as in the transmission of stress signals upwards [11]. Studies have found that roots can sense water gradients and guide their growth or the formation of new lateral roots to increase water use efficiency [12,13]. The perception of osmotic signals by the roots is mainly reflected in three aspects: local osmotic imbalance, changes in membrane tension, and disturbances in cell wall integrity. The conserved histidine kinase (*HK*) gene family in plants seems to act as the main osmosensors, but there is still controversy [14]. Changes in membrane tension caused by osmotic imbalance can be sensed by membrane mechanosensors, such as OSCA1, MSL, PIEZO, etc. [15]. The CrRLK family of receptor-like kinases (such as THE) sense the state of the cell wall, and their activation leads to cell wall reinforcement [16]. In the initial stages of signal transduction, the signal transmission network, which is composed of calcium ions and calcium-decoding proteins, facilitates rapid signal transmission and amplification. Moreover, cytosolic and nucleosolic calcium signals exhibit distinct responses to osmotic stress and function independently of each other [17]. Additionally, signals originating from the roots employ a diverse range of long-distance signaling mechanisms, including hydraulic signals, electrical signals, sugars, plant hormones, nucleic acids, proteins, and small peptides, to regulate shoot growth in response to salt and osmotic stresses [11]. Additionally, research has found that lncRNAs play a role in plants’ responses to salt stress and osmotic stress. In Arabidopsis, *npc536* is induced by high salt stress, and its overexpressing mutants exhibit more developed root systems [18]. *DRIR* is an lncRNA induced by salt and drought, whose overexpression enhances the plant’s tolerance to salt and drought stress [19]. In cotton, the overexpression of *lncRNA973* increases the plant’s tolerance to salt stress, while its silencing weakens the salt stress tolerance [20]. However, systematic identification of Ca^2+^-regulated lncRNAs in plants is still rarely involved.

During the initial phase of our research, we successfully developed transgenic plants expressing fusion proteins comprising rat parvalbumin (PV) tagged with either a nuclear export sequence (*PV-NES*) or a nuclear localization sequence (*NLS-PV*). Utilizing these transgenic plants, we demonstrated that the increases in cytosolic calcium ([Ca^2+^]_cyt_) and nuclear calcium ([Ca^2+^]_nuc_) levels triggered by osmotic or salt stress are independent of each other [17]. Here, we performed transcriptome sequencing on Arabidopsis WT and two regional calcium signaling-deficient mutants under salt stress and osmotic stress. Subsequently, using bioinformatics techniques, we newly identified 688 lncRNAs based on the existing Arabidopsis lncRNA database. We then found that cytosolic and nucleosolic calcium signaling affect the response of lncRNAs and PCGs to salt stress and osmotic stress. Analysis of the target PCGs regulated by calcium-regulated lncRNAs revealed that calcium-regulated lncRNAs are involved in the regulation of root morphological shaping and cell wall composition under salt stress and osmotic stress, through both *cis*-regulation and *trans*-regulation. Overall, the research highlights the importance of lncRNAs in plant stress tolerance and the intricate interplay between calcium signaling and gene regulation in Arabidopsis.

## 2. Results

### 2.1. Transcriptome-Wide Profiles of lncRNAs in Response to Salt and Hyperosmolarity Stresses in Arabidopsis

10-day-old Arabidopsis seedlings of WT, cytosolic-localized parvalbumin (*PV-NES*), and nucleosolic-localized parvalbumin (*NLS-PV*) plants [21] were treated with 125 mM NaCl or 250 mM sorbitol to simulate salt stress and hyperosmolarity stress, with each treatment lasting for 0, 6, and 24 h, respectively. Subsequently, real-time quantitative PCR (RT-qPCR) experiments were performed to detect the expression of salt-stress marker gene *MYB2* and hyperosmolarity-stress marker gene *RD29A* in WT [17]. The results showed that, compared to 0 h, both salt and hyperosmolarity stress treatments for 6 or 24 h significantly increased the expression of both genes, indicating that the treatment was successful (Figure S1). Interestingly, we further found that after 24 h of salt and hyperosmotic treatments, the expression of *MYB2* and *RD29A* in *PV-NES* and *NLS-PV* mutants showed no significant difference from the wild type. However, the expression of *RD29A* was significantly lower in both mutants after 6 h of NaCl/sorbitol treatment compared to WT, and the expression of *MYB2* was significantly lower in the *PV-NES* mutant after 6 h of NaCl/sorbitol treatment compared to WT. These results indicate that calcium signals in different regions regulated different expression patterns of the same gene.

**Figure 1:**
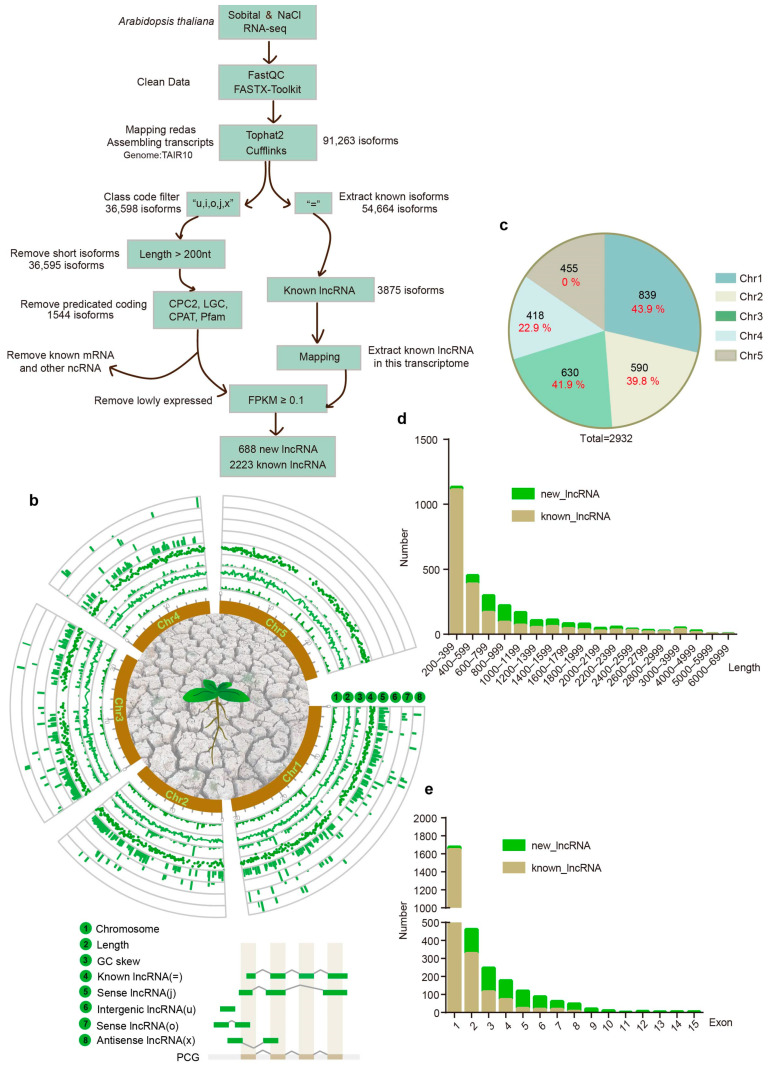
**Transcriptome-wide identification and characterization of long non-coding RNAs (lncRNAs) in *Arabidopsis thaliana*.** (**a**) The pipeline for the identification of lncRNAs in *Arabidopsis thaliana*. (**b**) Transcriptome-wide characterization of *Arabidopsis thaliana* lncRNAs. Circles 1 to 3 represent the distribution of different features of all lncRNAs on chromosomes. Circle 4 represents the distribution of known lncRNAs on chromosomes. Circles 5 to 8 represent the distribution of newly discovered lncRNAs from different sources on chromosomes. (**c**) Proportion of lncRNAs on different chromosomes in *Arabidopsis thaliana*. (**d**) Length distribution of new lncRNAs and known lncRNAs in *Arabidopsis thaliana*. (**e**) Exon number distribution of new lncRNAs and known lncRNAs in *Arabidopsis thaliana*.

Following RNA sequencing with a sequencing depth of 6 GB, we generated 54 RNA-seq datasets for lncRNA screening, as depicted in Figure 1a. The initial step involved rigorous cleaning and assembly of the transcriptome data, which led to the identification of 91,263 isoforms by aligning against the Arabidopsis TAIR10 database, serving as our reference genome. Within this database, 3875 lncRNAs have been previously identified. Employing an advanced lncRNA screening protocol, we conducted a comprehensive analysis to uncover novel lncRNAs. After filtering based on length, coding potential prediction, and the exclusion of known mRNAs and other ncRNAs, we selected isoforms with an FPKM value of at least 0.1 across any group, resulting in the identification of 688 novel lncRNAs encompassing 449 loci (Table S1). Among these, 538 lncRNAs were categorized as “j”, indicating potential new isoforms. The remaining lncRNA types were less frequent, with 55 “u” types, 48 “o” types, and 47 “x” types. Additionally, we screened for known lncRNAs that were expressed under NaCl and sorbitol stress conditions for further analysis, identifying 2223 known lncRNAs (Table S1). The chromosomal distribution and fundamental characteristics of Arabidopsis lncRNAs under salt and hyperosmotic stress are illustrated in the transcriptome-wide profiles (Figure 1b). These lncRNAs were distributed across all five Arabidopsis chromosomes, with a notable concentration on Chromosome 1 (Chr1), representing 28.6% of the total. It is noteworthy that the newly identified lncRNAs significantly increased the number of lncRNAs on nearly every chromosome. For instance, Chr1 exhibited a substantial 43.9% increase in lncRNA counts. However, no new lncRNAs were detected on Chromosome 5 (Chr5) (Figure 1c). Regarding sequence length distribution, lncRNAs were predominantly found in the 200–399 kb range, with a sparse presence beyond 5000 kb. The newly identified lncRNAs significantly expanded the representation in the 800–1200 bp range (Figure 1d, Table S2). In terms of exon number distribution, the overall pattern was characterized by a low number of exons, yet the newly screened lncRNAs exhibited a trend toward a higher number of exons, with the maximum reaching 15 (Figure 1e, Table S3). Lastly, there was no significant variation observed in the GC content and GC skew of the lncRNAs.

### 2.2. DElncRNAs and DEPCGs Respond to Cytosolic and Nucleosolic Calcium Signals in Different Ways in Terms of Quantity and Expression Level

**Figure 2:**
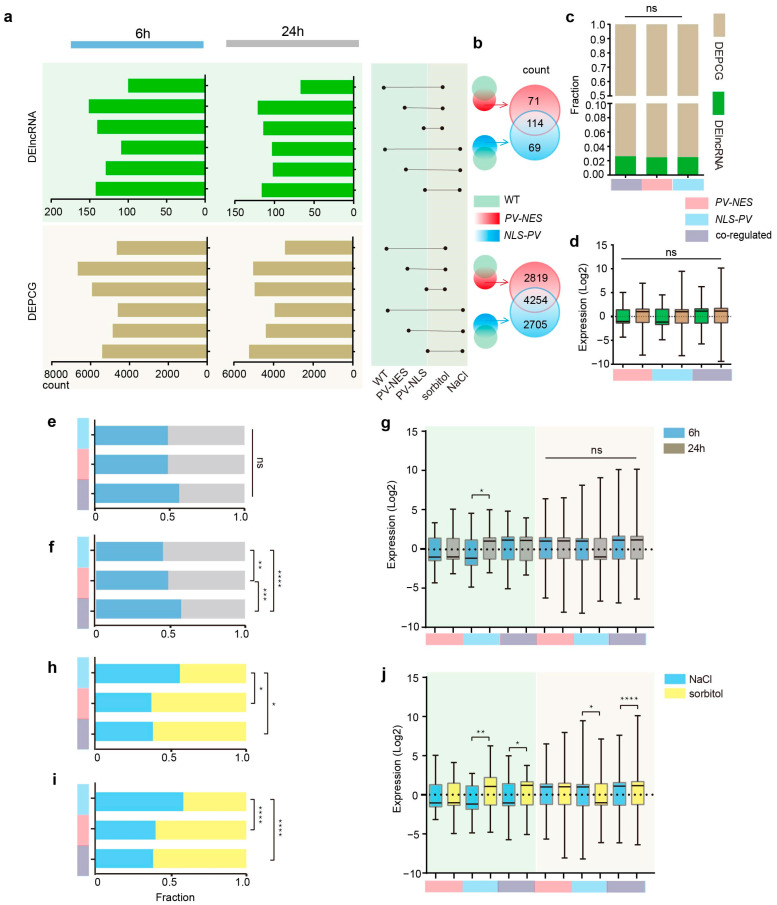
**Comparison of different stress-responsive lncRNAs and PCGs.** (**a**) The number of DElncRNAs and DEPCGs in response to NaCl or sorbitol for 6 h or 24 h in WT, *PV-NES* mutants, and *NLS-PV* mutants. (**b**) The number of DElncRNAs and DEPCGs regulated by cytosolic or nucleosolic calcium. (**c**) Fraction of DElncRNAs and DEPCGs regulated by [Ca^2+^]_cyt_, [Ca^2+^]_nuc_, both [Ca^2+^]_cyt_ and [Ca^2+^]_nuc_. (**d**) Expression of [Ca^2+^]_cyt_ or [Ca^2+^]_nuc_ or [Ca^2+^]_cyt_ and [Ca^2+^]_nuc_ co-regulated DElncRNAs and DEPCGs. (**e**) Fraction of 6 h-responsive and 24 h-responsive DElncRNAs. (**f**) Fraction of 6 h-responsive and 24 h-responsive DEPCGs. (**g**) Expression of 6 h-responsive and 24 h-responsive calcium-regulated DElncRNAs and DEPCGs. (**h**) Fraction of salt stress-responsive and hyperosmolarity stress-responsive calcium-regulated DElncRNAs. (**i**) Fraction of salt stress-responsive and hyperosmolarity stress-responsive calcium-regulated DEPCGs. (**j**) Expression of salt stress-responsive and hyperosmolarity stress-responsive calcium-regulated DElncRNAs and DEPCGs. Expression levels in each sample are computed in log2 (fold change). (* *p* < 0.05, ** *p* < 0.01, *** *p* < 0.001, **** *p* < 0.0001, two-sided Fisher’s exact test, two-sided Mann–Whitney *U* test).

To elucidate the intricate interplay between lncRNAs and PCGs in response to cytosolic and nucleosolic calcium signals, we conducted a differential expression analysis across 18 distinct conditions. We systematically paired these conditions randomly and selected pairs of interest based on plant material treated with NaCl or sorbitol for either 6 or 24 h, in comparison to the corresponding 0 h treatment. We designated lncRNAs and PCGs exhibiting |log2 fold change (FC)| ≥ 1, *p*-value ≤ 0.05, and a q-value ≤ 0.05 as differentially expressed genes, termed differentially expressed lncRNAs (DElncRNAs) and differentially expressed protein-coding genes (DEPCGs), respectively (Figure 2a, Tables S4 and S5).

In the WT, we identified 109 and 100 DElncRNAs in response to 6 h treatments and 103 and 67 DElncRNAs in response to 24 h NaCl or sorbitol treatments, respectively. In *PV-NES* plants, the numbers were 129 and 151 DElncRNAs for 6 h treatments and 102 and 121 for 24 h treatments; for *NLS-PV* mutants, we found 142 and 140 DElncRNAs for 6 h treatments and 116 and 114 for 24 h treatments, respectively. Our findings revealed that, relative to the WT, mutants exhibited a heightened mobilization of DElncRNAs under both stress conditions, with a particularly pronounced effect under sorbitol treatment, where the increase was nearly twofold. This suggested that cytosolic and nucleosolic calcium signals may exert an inhibitory influence on the stress response of lncRNAs, potentially accounting for our ability to identify novel lncRNAs on an existing foundation.

To delineate the regulatory effects of regional calcium signals on DElncRNAs and DEPCGs under salt and hyperosmotic stresses, we selected DElncRNAs and DEPCGs from the *PV-NES/NLS-PV* groups, excluding those that were also present in the wild type under each respective treatment condition. This approach enabled us to isolate differentially expressed genes (DEGs) caused by the blockade of cytosolic calcium ([Ca^2+^]_cyt_) or nucleosolic calcium ([Ca^2+^]_nuc_), and the intersection between these two sets revealed DEGs caused by the blockade of both [Ca^2+^]_cyt_ and [Ca^2+^]_nuc_ (Figure 2b). Our analysis identified 71 DElncRNAs and 2819 DEPCGs regulated exclusively by [Ca^2+^]_cyt_, 69 DElncRNAs and 2705 DEPCGs regulated exclusively by [Ca^2+^]_nuc_, and 114 DElncRNAs and 4254 DEPCGs regulated by both [Ca^2+^]_cyt_ and [Ca^2+^]_nuc_ (Figure 2b).

We further examined the influence of regional calcium signals on the regulation of DEGs in terms of both the quantity and expression levels of DEGs. We observed no significant impact of distinct regional calcium signals on the quantity ratio and expression levels of DElncRNAs and DEPCGs (Figure 2c,d). Concerning the duration of stress, the number of DElncRNAs was not significantly modulated by regional Ca^2+^, whereas their expression levels were more markedly regulated by [Ca^2+^]_nuc_ alone, with a pronounced increase at 24 h compared to 6 h (Figure 2e,g). In contrast, the number of DEPCGs was significantly influenced by regional calcium signals, with notable differences under all three calcium signal patterns; in the 6 h treatment, DEPCGs were primarily co-regulated by [Ca^2+^]_cyt_ and [Ca^2+^]_nuc_, with [Ca^2+^]_nuc_ regulation becoming predominant as the treatment duration increased; their expression levels, however, were not influenced by different calcium signal patterns (Figure 2f,g).

In response to distinct treatments, DElncRNAs and DEPCGs exhibited a relatively similar pattern of regional calcium signal response (Figure 2h–j). For DElncRNAs and DEPCGs regulated solely by [Ca^2+^]_nuc_, there was a greater number of responses to NaCl than to sorbitol in terms of quantity, while the expression levels of DElncRNAs were higher under NaCl than under sorbitol, and the opposite was true for DEPCGs. For those DElncRNAs and DEPCGs regulated exclusively by [Ca^2+^]_cyt_, their responses to sorbitol were more numerous than to NaCl, with no significant difference in expression levels between the two treatments. For DElncRNAs and DEPCGs co-regulated by [Ca^2+^]_cyt_ and [Ca^2+^]_nuc_, there was a significantly greater response to sorbitol than to NaCl in both quantity and expression levels.

In conclusion, our findings reveal that different regional calcium signals exert distinct regulatory patterns on DElncRNAs and DEPCGs and exhibit varied response patterns to salt stress and hyperosmotic stress. These insights contribute to a deeper understanding of the complex regulatory networks involving calcium signaling in plant stress responses.

### 2.3. Co-Expression Network Reveals the Role of Calcium-Regulated lncRNAs and PCGs in Response to Salt Stress and Hyperosmotic Stress

To dissect the intricate roles of lncRNAs and PCGs modulated by cytosolic and nucleosolic calcium signals within salt stress and hyperosmolarity stress signaling pathways, we conducted a comprehensive screening of the transcriptomes for genes that respond to NaCl or sorbitol treatments and are under the influence of calcium signals within the nucleus or cytoplasm. Our analysis revealed 2193 lncRNAs and 36,404 PCGs responsive to NaCl and regulated by [Ca^2+^]_cyt_, 2331 lncRNAs and 37,712 PCGs responsive to sorbitol and regulated by [Ca^2+^]_cyt_, 2239 lncRNAs and 37,179 PCGs responsive to NaCl and regulated by [Ca^2+^]_nuc_, and 2273 lncRNAs and 36,962 PCGs responsive to sorbitol and regulated by [Ca^2+^]_nuc_ (Table S6).

**Figure 3:**
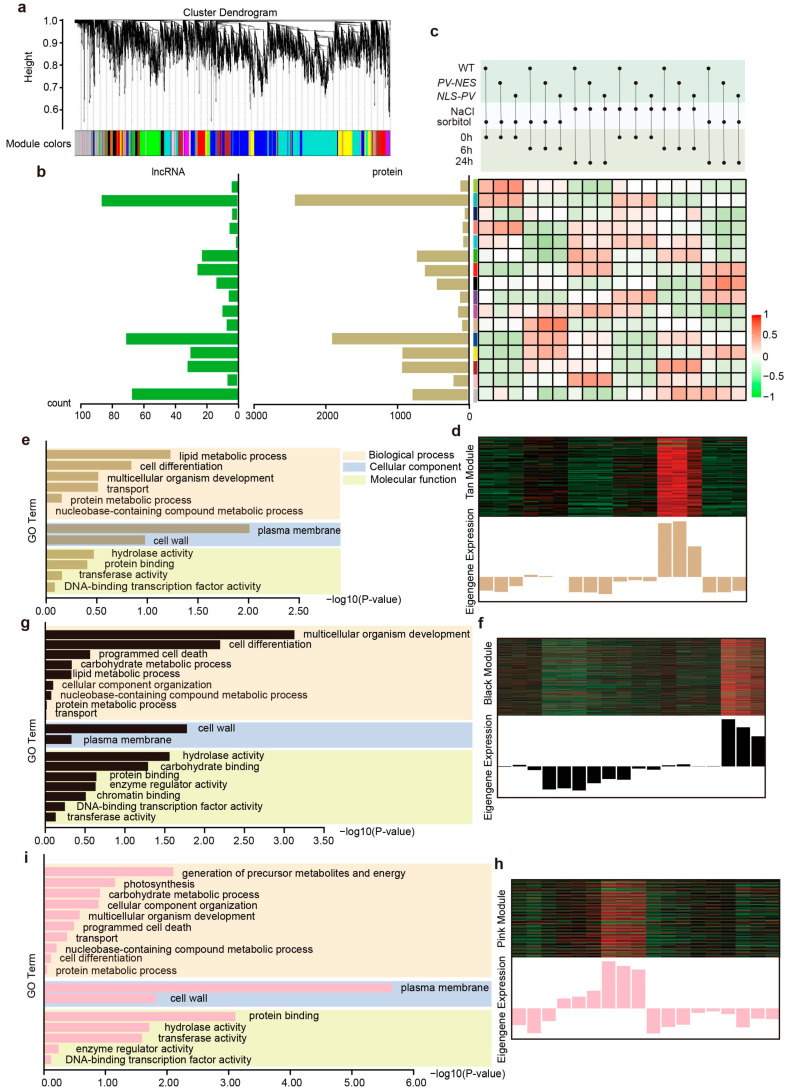
WGCNA analysis of *trans*-regulated lncRNAs and their PCGs in *Arabidopsis thaliana* in response to salt stress and hyperosmolality stress. (**a**) Hierarchical cluster tree and color bands showing the 16 modules by WGCNA (*n* = 10,000). (**b**) The distribution of lncRNAs and PCGs number in each module. (**c**) The correlation analysis between module and trait. (**d**) Eigengene expression profile for the “Tan” module in *PV-NES* and *NLS-PV* mutants in response to sorbitol 6 h treatment. (**e**) Terms of GO enrichment analysis among PCGs and their *trans*-regulated lncRNAs of the “Tan” module. (**f**) Eigengene expression profile for the “Black” module in *PV-NES* mutants in response to sorbitol 24 h treatment. (**g**) Terms of GO enrichment analysis among PCGs and their *trans*-regulated lncRNAs of the “Black” module. (**h**) Eigengene expression profile for the “Pink” module in *PV-NES* mutants in response to NaCl 24 h treatment. (**i**) Terms of GO enrichment analysis among PCGs and their *trans*-regulated lncRNAs of the “Pink” module.

We then put all the calcium-regulated lncRNAs and PCGs on the Weighted Gene Co-expression Network Analysis (WGCNA) website. After a series of parameter settings, 391 Ca^2+^-regulated lncRNAs and 9609 Ca^2+^-regulated PCGs implicated in salt stress and hyperosmolarity stress were selected, with the objective of identifying modules of lncRNAs and PCGs that exhibit a high degree of association with regional calcium signal responses to these stressors (Table S7). Through hierarchical clustering, we identified 16 highly correlated modules (Figure 3a), with the distribution of lncRNAs and PCGs within each module illustrated in Figure 3b. Notably, the “Turquoise”, “Blue”, and “Grey” modules contained a significantly higher number of clustered lncRNAs and PCGs compared to other modules. Although the number of lncRNAs in each module was relatively sparse, with the “Cyan” module containing a single lncRNA, the abundance of co-expressed PCGs in each module was substantial. This allows us to infer the functional roles of lncRNAs through *trans*-regulation by leveraging the biological functions of their co-expressed PCGs.

Subsequently, we focused on modules that were specifically regulated by cytosolic or nucleosolic calcium in response to NaCl or sorbitol, namely the “Tan” (*p*-value = 0.0078), “Black” (*p*-value = 0.011), and “Pink” (*p*-value = 0.03) modules (Figure 3c), for further investigation. These modules were selected based on their statistical significance and potential regulatory roles in stress response pathways, providing a foundation for deeper exploration into the molecular mechanisms underlying calcium-mediated stress signaling in plants.

**Figure 4:**
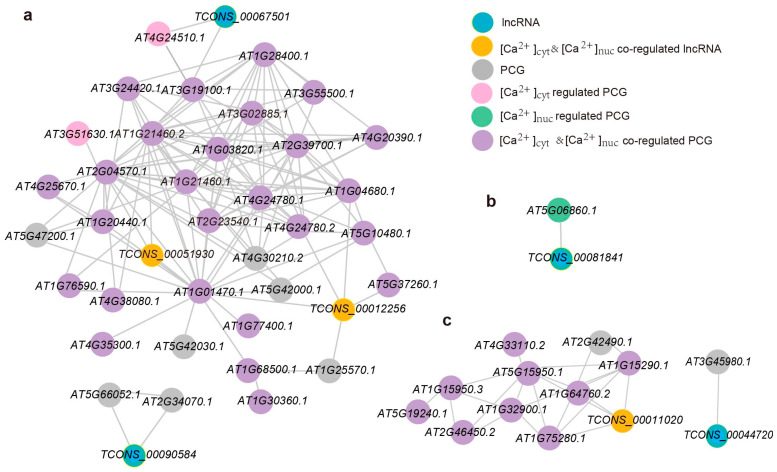
**Co-expression network of lncRNAs and their *trans*-regulated PCGs.** (**a**) The co-expression network of the “Tan” module (weight threshold = 0.2). (**b**) The co-expression network of the “Black” module (weight threshold = 0.2). (**c**) The co-expression network of the “Pink” module (weight threshold = 0.1). Only networks related to lncRNA are displayed.

In the “Tan” module, we observed a significant upregulation in the expression levels of lncRNAs and PCGs following a 6 h sorbitol treatment, surpassing those of other treatment and time groups (Figure 3d). Relative to the wild-type (WT) group, the *NLS-PV* group exhibited comparatively reduced expression levels, while the *PV-NES* group maintained expression levels akin to the WT. Subsequently, a co-expression network was constructed for the lncRNAs and PCGs within the “Tan” module, with a soft threshold power set to 0.2, yielding 41 nodes and 141 edges (Figure 4a). A Gene Ontology (GO) functional enrichment analysis of the PCGs in this network indicated a predominant distribution in the plasma membrane and cell wall, with involvement in lipid metabolic processes, cell differentiation, multicellular organism development, and other biological processes, as well as molecular functions such as hydrolases and protein binding (Figure 3e). Four lncRNAs were identified in the co-expression network, with *TCONS_00051930* ([Ca^2+^]_cyt_ and [Ca^2+^]_nuc_ co-regulated sorbitol-responsive lncRNA), *TCONS_00012256* ([Ca^2+^]_cyt_ and [Ca^2+^]_nuc_ co-regulated sorbitol-responsive lncRNA), and *TCONS_00067501* being part of the largest co-expression network (Figure 4a). Three ([Ca^2+^]_cyt_ and [Ca^2+^]_nuc_ co-regulated sorbitol-responsive PCGs, *AT2G04570.1* (*GGL14*), *AT1G21460.2* (*SWEET1*), and *AT1G01470.1* (*LEA14*), were the first neighbors of *TCONS_00051930*, suggesting a higher degree of functional association (Figure 4a). Apart from *AT1G01470.1*, *TCONS_00012256* had four additional first neighbors, including *AT4G24780.2* (*PLL19*), *AT1G04680.1* (*PLL26*), *AT5G37260.1* (*CIR1*), and *AT1G25570.1* (*LLR1*). *TCONS_00067501* had only two first neighbors, *AT4G24510.1* (*CER2*) and *AT3G19100.1* (*TAGK2*). *TCONS_00090584* formed a small network with *AT5G66052.1* and *AT2G34070.1* (*TBL37*).

In the “Black” module, which is associated with sorbitol treatment, the expression levels of lncRNAs and PCGs after a 24 h treatment were markedly higher than those of other treatment methods and times. Compared to the WT group, both the *PV-NES* and *NLS-PV* groups showed relatively lower expression levels, with the *NLS-PV* group exhibiting even more reduced levels (Figure 3f). We constructed a co-expression network for the lncRNAs and PCGs in the “Black” module with a power weight of 0.2, resulting in 56 nodes and 137 edges, which included only one lncRNA, *TCONS_00081841*. A GO functional enrichment analysis of the PCGs in this network revealed that, in comparison to the 6 h treatment, the 24 h treatment group exhibited distinct characteristics: in cellular components, the 24 h group was more abundant in the cell wall, followed by the plasma membrane; in biological processes, multicellular organism development and cell differentiation became the main focus, with the emergence of programmed cell death and carbohydrate metabolic processes; in molecular functions, carbohydrate binding, enzyme regulator activity, and chromatin binding were newly identified (Figure 3g). *TCONS_00081841* formed a network exclusively with the [Ca^2+^]_nuc_-regulated PCG-*AT5G06860.1* (*PGIP1*) (Figure 4b). PGIP1 is known to inhibit the function of cell wall pectin-degrading enzymes, such as those produced by fungal pathogens, and its knockout mutant exhibited severe damage in the root tip under low Ca^2+^ and low pH conditions [22].

The “Pink” module, associated with NaCl treatment, showed significantly higher expression levels of lncRNAs and PCGs after a 24 h NaCl treatment compared to other treatment methods and time groups. Relative to the WT group, both the *PV-NES* and *NLS-PV* groups displayed lower expression levels, with the *NLS-PV* group showing even less expression (Figure 3h). In this co-expression network, with a power weight of 0.1, we identified 85 nodes and 131 edges, including two lncRNAs, *TCONS_00011020* and *TCONS_00044720*. A GO functional enrichment analysis of the PCGs in this network revealed that, in contrast to sorbitol treatment, PCGs after 24 h of NaCl treatment were primarily distributed in the plasma membrane, followed by the cell wall, and were involved in biological processes such as the generation of precursor metabolites and energy, as well as photosynthesis, with molecular functions reflecting protein binding, hydrolase activity, and transferase activity (Figure 3i). *TCONS_00011020*, an lncRNA co-regulated by [Ca^2+^]_cyt_ and [Ca^2+^]_nuc_, is located in the main network and is directly adjacent to *AT1G15290.1* (*REC3*), *AT5G15950.1* (*SAMDC2*), *AT1G64760.2* (*ZERZAUST*), and *AT1G75280.1*, all of which are PCGs co-regulated by [Ca^2+^]_cyt_ and [Ca^2+^]_nuc_ (Figure 4c). *TCONS_00044720*, on the other hand, forms a separate network with *AT3G45980.1* (*H2B*), which is involved in regulating chromatin structure and function [23].

### 2.4. Ca^2+^-Regulated lncRNAs and Their Potential Cis-Regulated Target PCGs in Omostic Stress

**Figure 5:**
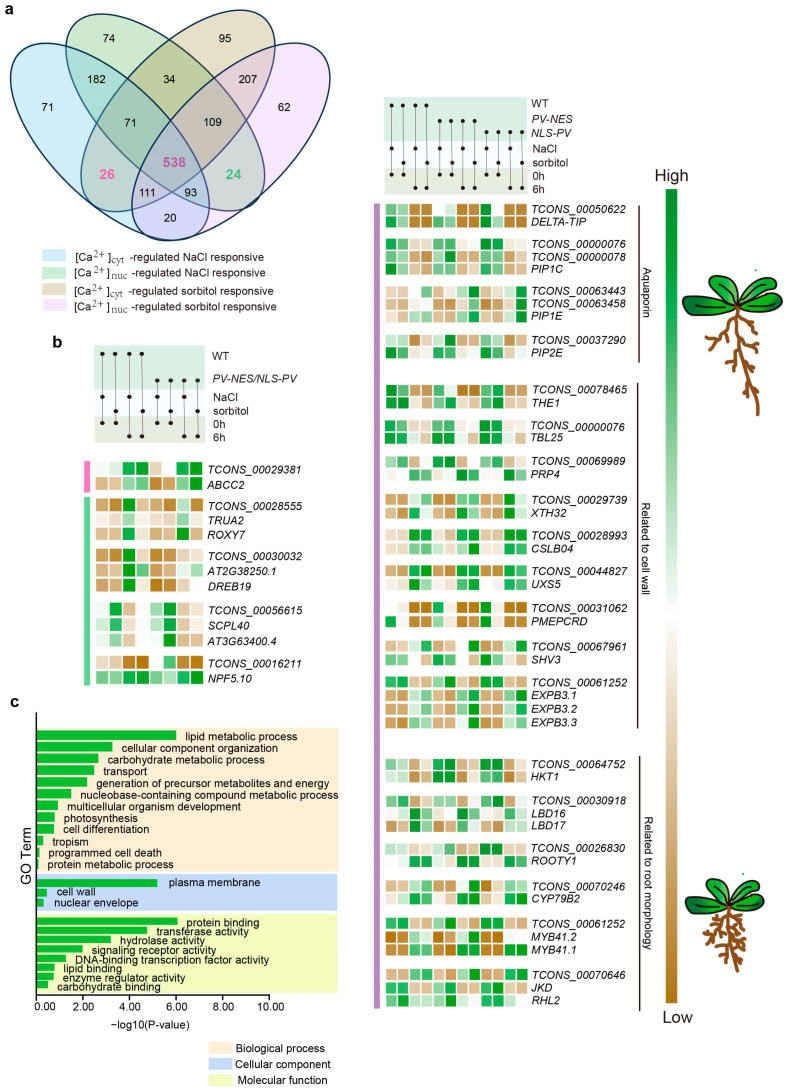
**Functions of calcium-regulated osmotic stress-responsive lncRNA for 6 h treatment.** (**a**) Selection of 6 h osmotic stress-responsive lncRNAs totally regulated by [Ca^2+^]_cyt_, [Ca^2+^]_nuc_ or both [Ca^2+^]_cyt_ and [Ca^2+^]_nuc_ in *Arabidopsis thaliana*. (**b**) Expression pattern of *cis*-regulated target PCGs of calcium-regulated lncRNAs. (**c**) GO enrichment analysis at different levels of [Ca^2+^]_cyt_ and [Ca^2+^]_nuc_ co-regulated osmotic stress-responsive *cis*-regulated target PCGs.

**Figure 6:**
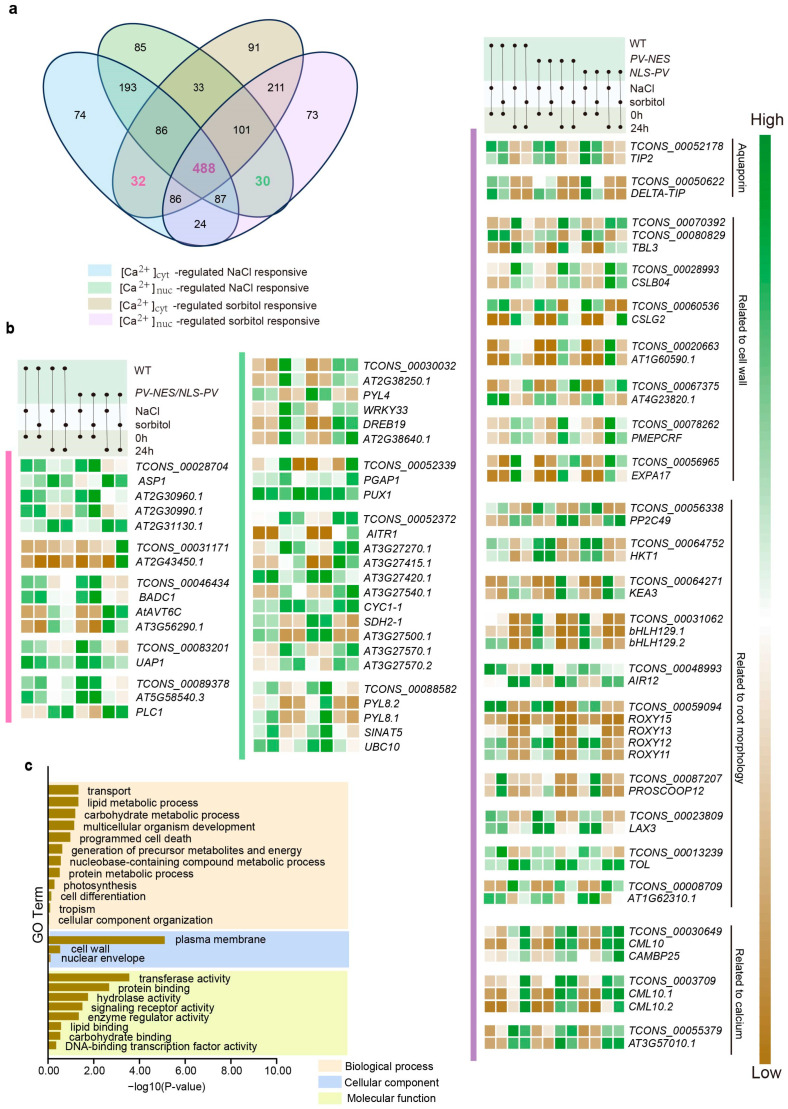
**Functions of calcium-regulated osmotic stress-responsive lncRNA for 24 h treatment.** (**a**) Selection of 24 h osmotic stress-responsive lncRNAs totally regulated by [Ca^2+^]_cyt_, [Ca^2+^]_nuc_ or both [Ca^2+^]_cyt_ and [Ca^2+^]_nuc_ in *Arabidopsis thaliana*. (**b**) Expression pattern of *cis*-regulated target PCGs of calcium-regulated lncRNAs. (**c**) GO enrichment analysis at different levels of [Ca^2+^]_cyt_ and [Ca^2+^]_nuc_ co-regulated osmotic stress-responsive *cis*-regulated target PCGs.

*Cis*-regulation of target PCGs by lncRNAs is a pivotal mechanism of lncRNA function, which is largely contingent upon the genomic position of lncRNA transcription sites and their proximity to target PCGs within the linear genome [3]. Current research suggests that *cis*-regulated lncRNAs are capable of activating, repressing, or otherwise modulating the expression of target genes [24]. Stress induced by either NaCl or sorbitol treatment can be categorized into two types: stress due to different ions or molecules and osmotic stress, with osmotic stress being the common denominator. To simplify the complexity of calcium signal responses to these different treatments, we focused on lncRNAs regulated by [Ca^2+^]_cyt_, [Ca^2+^]_nuc_, and both [Ca^2+^]_cyt_ and [Ca^2+^]_nuc_ in response to osmotic stress. Specifically, we examined 26 [Ca^2+^]_cyt_-regulated, 24 [Ca^2+^]_nuc_-regulated, and 538 [Ca^2+^]_cyt_ and [Ca^2+^]_nuc_ co-regulated lncRNAs after 6 h of treatment (Figure 5a), as well as 32 [Ca^2+^]_cyt_-regulated, 30 [Ca^2+^]_nuc_-regulated, and 488 [Ca^2+^]_cyt_ and [Ca^2+^]_nuc_ co-regulated lncRNAs after 24 h of treatment (Figure 6a). Employing Bedtools and utilizing the Arabidopsis TAIR10 database as the reference genome, we identified potential *cis*-regulated target PCGs within a 100 kb window upstream and downstream of the selected lncRNA transcription sites [25]. For the 6 h treatment, we identified 3365 [Ca^2+^]_cyt_-regulated, 2834 [Ca^2+^]_nuc_-regulated, and 69,204 [Ca^2+^]_cyt_ and [Ca^2+^]_nuc_ co-regulated lncRNA-PCG pairs. For the 24 h treatment, we found 4289 [Ca^2+^]_cyt_-regulated, 3856 [Ca^2+^]_nuc_-regulated, and 61,837 [Ca^2+^]_cyt_ and [Ca^2+^]_nuc_ co-regulated lncRNA-PCG pairs. Subsequently, we calculated the Pearson correlation coefficient for all lncRNA-PCG pairs, screening for lncRNA-PCG pairs with |PCC| ≥ 0.9 and *p* value < 0.05. Ultimately, for the 6 h treatment, we obtained one [Ca^2+^]_cyt_-regulated, four [Ca^2+^]_nuc_-regulated, and 115 [Ca^2+^]_cyt_ and [Ca^2+^]_nuc_ co-regulated lncRNA-PCG pairs (Table S8); for the 24 h treatment, we obtained five [Ca^2+^]_cyt_-regulated, four [Ca^2+^]_nuc_-regulated, and 152 [Ca^2+^]_cyt_ and [Ca^2+^]_nuc_ co-regulated lncRNA-PCG pairs (Table S8).

In the 6 h treatment, *ABCC2*, the sole [Ca^2+^]_cyt_ -related *cis*-regulated PCG, plays a role in the transport of anthocyanins and other flavonoids (Figure 5b) [26]. The [Ca^2+^]_nuc_ -related *cis*-regulated PCGs include transcription factors such as *ROXY7*, *AT2G38250.1*, and *DREB19*, enzymes related to protein synthesis including *TRUA2*, *SCPL40*, and *AT3G63400.4*, as well as the transporter *NPF5.10* (Figure 5b). The 115 [Ca^2+^]_cyt_ and [Ca^2+^]_nuc_ co-regulated lncRNAs had 458 *cis*-regulated target PCGs, and a GO analysis was performed on these PCGs (Figure 5c). These PCGs were primarily associated with lipid metabolic processes, cellular component organization, carbohydrate metabolic processes, and transport, among others. Notably, we identified several aquaporin (*AQP*) genes, including *DELTA-TIP*, *PIP1C*, *PIP1E*, and *PIP2E* (Figure 5b). Studies on the role of aquaporins during early stages of lateral root development have shown that most aquaporin genes are repressed during lateral root formation in an auxin-dependent manner [27]. In our study, we observed decreased expression levels of *DELTA-TIP*, *PIP1C*, and *PIP2E*, while *PIP1E* expression was increased (Figure 5b). We also found PCGs involved in the composition and modification of the cell wall, such as *THE1*, *TBL25*, *RUBY*, and *PRP4*, etc. (Figure 5b). Additionally, numerous PCGs related to root morphology and development, particularly lateral root development, were identified, including *HKT1*, *LBD* family genes, *ROOTY1*, etc. (Figure 5b).

In the 24 h treatment, a few PCGs *cis*-regulated by [Ca^2+^]_cyt_-regulated lncRNAs of unknown function were observed, while the rest were involved in processes such as the synthesis of fatty acids associated with the plasma membrane (*BADC1*) and amino acids related to osmotic solutes (*AtAVT6C*, *UAP1*, *PLC1*). For [Ca^2+^]_nuc_-regulated lncRNAs, their *cis*-regulated PCGs were predominantly involved in the ABA signaling pathway, including ABA sensors (*PYL4*, *PYL8*) and feedback regulators (*AITR1*). We also noted the regulation of *TCONS_00030032*-*DREB19* in this context. The 152 [Ca^2+^]_cyt_ and [Ca^2+^]_nuc_ co-regulated lncRNAs had 402 *cis*-regulated target PCGs, and a GO analysis was performed on these PCGs as well (Figure 6c). Compared to the 6 h treatment, these PCGs were more evenly distributed and participated in a broader range of biological processes, with the top four being transport, lipid metabolic process, carbohydrate metabolic process, and multicellular organism development. Here, we again identified *AQPs* (*TIP2* and *DELTA-TIP*) involved in osmotic stress, which were located in the tonoplast (Figure 6b). Additionally, *HKT1* was identified, along with its negative regulator *PP2C49*, and *KEA3*, another potassium channel. There were also PCGs related to root growth, such as *bHLH129*, the *ROXY* family genes, *PROSCOOP12*, and *AIR12*, etc., and PCGs associated with the cell wall included *TBL3*, *CLSB04*, *CLSG2*, etc. (Figure 6b). It is noteworthy that calcium-related PCGs, such as calcium decoding protein genes (*CML10* and *CAMBP25*) and calcium-dependent protein genes (*AT3G57010.1* and *VLN3*), were prominently featured (Figure 6b).

## 3. Discussion

### 3.1. Regulation of Plant Cell Wall Strength by lncRNAs in Response to Salt Stress and Osmotic Stress

Plant cell walls, which are primarily composed of polysaccharide polymers, lignin, and a subset of structural proteins, serve as the protective envelope of plant cells, maintaining their shape and providing mechanical strength [28]. These walls are conventionally divided into three distinct regions: the middle lamella, the primary cell wall, and the secondary cell wall. The primary cell wall is predominantly made up of cellulose, hemicelluloses, and pectins that function as cross-linking agents [29]. Xyloglucan, a major class of hemicellulose polymers, binds to cellulose fibers, a process regulated by xyloglucan endotransglucosylase/hydrolases (XTHs) and expansins [30,31,32]. Early observations in plants under osmotic stress have noted a significant upregulation in the expression of XTHs and/or expansins, with transgenic plants overexpressing these proteins exhibiting enhanced salt tolerance [30,33,34]. In our study, we observed a marked increase in the expression of *XTH32*, a member of the XTH family Group 3, under osmotic stress, particularly in the absence of cytosolic and nucleosolic calcium, suggesting a *cis*-regulation by the calcium-responsive lncRNA *TCONS_00029739*. Additionally, we identified significant upregulation of α-expansins (*EXPAs*), such as *EXPA17*, involved in cellulose-cellulose linkages, and three transcripts of β-expansins (*EXPBs*), such as *EXPB3*, involved in xyloglucan. The expression of *EXPB3* is negatively regulated by *TCONS_00061252*, with increased expression in the absence of regional calcium signals, while *EXPA17*, regulated by *TCONS_00056965*, shows a less pronounced increase in expression under similar conditions. Similarly, the expression of cellulose synthesis-related enzymes, such as *CSLB04*, *CSLG2*, and *SHV3*, was significantly upregulated under the regulation of calcium-responsive lncRNAs, implying an increase in cell wall rigidity [35].

Homogalacturonan (HG), the most abundant pectin type, is highly methyl-esterified and synthesized in the Golgi apparatus before being transported to the cell wall, where it is demethylated by pectin methyl-esterases [36]. The modification and increased content of pectin are beneficial for plant growth under salt stress and drought. Comparative studies between drought-resistant or salt-tolerant varieties and sensitive varieties of wheat and soybean have revealed higher pectin content in resistant varieties, which is advantageous for root growth [37,38]. We identified a multitude of pectin-related enzyme genes among the lncRNA target PCGs. Among the PCGs regulated by lncRNA *trans*-regulation, PGIP1 was found to inhibit the function of cell wall pectin-degrading enzymes [22] (Figure 4a). In the context of *cis*-regulation, the expression of pectin methyl-esterase inhibitor *PMEPCRD* was significantly downregulated, while the expression of *PMEPCRF* increased with prolonged treatment, and the two exhibited differential responses to distinct calcium signals (Figure 5b and Figure 6b). *PLL19* and *PLL26*, encoding pectate lyases, were highly associated with *TCONS_00012256* (Figure 4a). Pectin lyase-like family members *AT1G60590.1* and *AT4G23820.1* displayed opposing response patterns, suggesting a dynamic regulation of pectin content by lncRNAs (Figure 6c). Furthermore, lncRNAs also regulate the expression of enzymes related to the synthesis of precursor products for cell wall components, such as *UXS5*, *ZERZAUST*, and UDP-Glycosyltransferase gene, providing material assurance for the strengthening of the cell wall. In addition, lncRNAs regulate the genes related to the accumulation of secondary wall cellulose, the TRICHOME BIREFRINGENCE-LIKE (TBL) gene family, including *TBL3*, *TBL25*, and *TBL37* (Figure 5b and Figure 6b).

Thus, it is highly plausible that lncRNAs play a pivotal role in fortifying the mechanical properties of the plant cell wall, thereby enhancing the plant’s tolerance to salt stress and osmotic stress. This enhancement is mediated through the regulation of various enzymes and proteins involved in the synthesis, modification, and organization of cell wall components, such as pectin, cellulose, and hemicelluloses. By modulating the expression and activity of these key players, lncRNAs can influence the cell wall’s structure and functionality, which in turn affects the plant’s ability to withstand adverse environmental conditions.

### 3.2. Calcium-Regulated lncRNAs Are Involved in the Regulation of Root Morphology Under Osmotic Stress in Arabidopsis

The plant root system is instrumental in anchoring the plant, absorbing nutrients and water, and facilitating their transport during plant growth [11]. However, under salt stress and osmotic stress, the growth and development of the root system are impeded, leading to alterations in the length of the primary root and the density of lateral roots and root hairs [39,40]. Research has established that salt-tolerant plant varieties maintain a high K^+^/Na^+^ ratio in their roots, with a positive correlation existing between this ratio and the root branching zone [41,42]. HKT1, a sodium transporter protein, is known to accumulate Na^+^ ions in the roots of seedlings when highly expressed [14]. PP2C49, a negative regulator of HKT1 activity, plays a crucial role in determining the systemic allocation of Na^+^ during salt stress [14]. In our study, we identified that *TCONS_00064752 cis*-regulates the downregulation of *HKT1* expression (Figure 5b and Figure 6b), while *TCONS_00056338 cis*-regulates the upregulation of *PP2C49* expression (Figure 6b). Additionally, KEA3, localized to the thylakoid membrane, facilitates proton efflux from the thylakoid lumen through proton/potassium antiport [43]. We found that the expression of *KEA3* is downregulated by *TCONS_00064271* in response to osmotic stress (Figure 6b). Consequently, lncRNAs appear to regulate the K^+^/Na^+^ ratio in roots, resulting in reduced Na^+^ and increased K^+^, which may contribute to enhanced lateral root development.

Furthermore, various families of transcription factors are pivotal in the root’s stress response, including the Lateral Organ Boundaries Domain (LBD) family proteins, bHLH transcription factors, and MYB family proteins [44,45,46]. The triple mutant of *LBD16*, *LBD29*, and *LBD18* exhibits significant defects in lateral root formation by downregulating the expression levels of D-type cyclins (*CYCDs*), PLETHORA (*PLT*), and PIN-FORMED (*PIN*) in the auxin signaling pathway [47]. The bHLH transcription factor family member bHLH129 acts as a transcriptional repressor of the ABA response, and its overexpression promotes root elongation [45]. Under NaCl and sorbitol treatment, we observed that lncRNAs upregulated the expression levels of *LBD16* and *LBD17* and downregulated the expression levels of two transcripts of *bHLH129*. On the other hand, the biosynthesis and deposition of suberin can prevent water loss during salt stress [48]. We also found that the transcription factor *MYB41*, involved in the biosynthesis and deposition of suberin [49], is a target gene of calcium-regulated lncRNA, and its expression increases under osmotic stress, particularly in the absence of cytosolic and nucleosolic calcium, suggesting that calcium signaling may play a negative regulatory role in its expression. Calcium-regulated lncRNAs also regulate a series of PCGs involved in lateral root and root hair formation, including upregulating the expression of *CYP79B2* [50], *ROOTY1* [51], *AIR12* [52], *TOL* [53], etc., and downregulating the expression of the *ROXY* family genes [54], *PROSCOOP12* [55], *LAX3* [56], etc., and may directly regulate *SAMDC2* [57], which is consistent with the phenotype of increased lateral root number and root hair under osmotic stress.

LncRNAs appear to play a central role in influencing the expression of genes that control the K^+^/Na^+^ ratio, root branching, and the development of lateral roots and root hairs. This molecular regulation is essential for plant survival under stress conditions, and understanding these mechanisms could inform strategies for developing plants with enhanced tolerance to environmental challenges.

## 4. Materials and Methods

### 4.1. Plant Materials and Treatment

The Arabidopsis thaliana Columbia ecotype utilized in this study were subjected to experimental protocols, with *PV-NES* and *NLS-PV* mutant materials previously constructed in our laboratory [17]. The seeds of Arabidopsis were surface-sterilized using a 75% ethanol solution and subsequently sown on 1/2 strength Murashige and Skoog (MS) medium. The plates were then subjected to stratification at 4 °C in the dark for 3 days to facilitate vernalization, followed by transfer to a growth chamber for a 7-day cultivation period under controlled environmental conditions: light intensity 120 μmol m^−2^s^−1^, temperature 21 °C, 50% relative humidity, and a photoperiod of 16 h light and 8 h darkness. Uniformly developed seedlings were selected and subjected to treatments with aqueous solutions of 125 mM NaCl or 250 mM sorbitol for durations of 0 h, 6 h, and 24 h, respectively. Upon completion of the treatment periods, the seedlings were carefully harvested, any surface moisture was gently removed, and the specimens were promptly stored in liquid nitrogen for further analysis.

### 4.2. RNA Extraction and Real-Time Quantitative PCR

RNA extraction of seedlings was conducted in a low-temperature setting, employing the Eastep^®^ Super Total RNA Extraction Kit (Promega, Madison, WI, USA) in accordance with the manufacturer’s protocol. Subsequently, 500 ng of RNA from each sample was subjected to reverse transcription to synthesize complementary DNA (cDNA), utilizing the First-Strand cDNA Synthesis SuperMix kit (TransScript, Beijing, China) for this procedure. The resulting cDNA was diluted to an optimal concentration and employed as a template for real-time quantitative polymerase chain reaction (RT-qPCR). The RT-qPCR reactions were executed using the Go Taq^®^ qPCR Master Mix kit (Promega, Madison, WI, USA), with primers specific for the genes MYB2 and RD29A, which were sourced from the study by Huang et al. [17], and actin as an internal reference.

### 4.3. RNA-Seq and Transcriptome Assembly

RNA sequencing (RNA-seq) was performed utilizing ANOROAD GENOME (Beijing, China)’s paired-end sequencing service, which is based on the Illumina sequencing platform, yielding 108 raw data files across 54 samples. The sequencing data were uploaded to the Genome Sequence Archive (Genomics, Proteomics, and Bioinformatics 2021) [58] in the National Genomics Data Center (Nucleic Acids Res 2022) [59], China National Center for Bioinformation/Beijing Institute of Genomics, Chinese Academy of Sciences (GSA: CRA020770) that are publicly accessible at https://ngdc.cncb.ac.cn/gsa (accessed on 6 December 2024). To ascertain the quality of the data for subsequent analyses, we utilized Fast QC (version 0.12.1, http://www.bioinformatics.babraham.ac.uk/projects/download.html#fastqc, accessed on 1 March 2023, with parameters -q 20 and -*p* 90) and Fast X-toolkit (version 0.0.14, http://hannonlab.cshl.edu/fastx_toolkit, accessed on 18 February 2022, with parameter -f 7) to filter out low-quality reads from the raw data and to eliminate the initial 7 nucleotides from each read. Following this, the Arabidopsis TAIR10 genome and corresponding annotation files were retrieved from The Arabidopsis Information Resource (https://www.arabidopsis.org/, accessed on 24 October 2000), and bowtie2 (version 2.5.4, https://sourceforge.net/projects/bowtie-bio/, accessed on 16 May 2024) was employed to construct the reference index. Thereafter, TopHat2 (version 2.2.1, https://ccb.jhu.edu/software/tophat/index.shtml, accessed on 23 February 2016) was used to map the cleaned reads from each sample to the TAIR10 reference genome. The alignment outcomes from TopHat2 served as the foundation for Cufflinks (version 2.2.1, https://mirrors.aliyun.com/macports/packages/cufflinks/, accessed on 5 May 2014) to compute the isoform-level FPKM (Fragments Per Kilobase of transcript, per Million mapped reads) values, relying on the reference genome’s annotation files, and to assemble the transcriptome.

### 4.4. Pipline of lncRNA Screening

The identification of lncRNAs was conducted following the established protocol described in reference [6]. The procedure can be succinctly outlined as follows:

(1) Transcript Filtering: Transcripts with class code types “i”, “o”, “j”, “u”, and “x” were selected from the assembled transcriptome.
(2) Length Filtering: Transcripts with lengths less than 200 nucleotides were excluded from further consideration.
(3) Coding Potential Prediction: The subsequent step involved the application of the software CPC2, LGC, CPAT, and Pfam to predict the protein-coding potential of the remaining transcripts, thereby facilitating the exclusion of those possessing coding capabilities.
(4) Blast Alignment: The transcripts that remained were aligned against a database of known mRNAs and other non-coding RNAs using Blast, with criteria set for high stringency (E-value < 10^ (−10), identity > 90%) to eliminate any matches.
(5) Expression Level Screening: The final step involved the assessment of expression levels, with transcripts required to exhibit an FPKM value of at least 0.1 in at least one sample to be considered. This threshold was crucial for the identification of novel lncRNAs. For known lncRNAs, the same expression level filter was applied to ensure that only those with significant expression were included in the subsequent analysis.

### 4.5. Basic Characteristics of lncRNAs

The fundamental characteristics of lncRNAs, which encompass their chromosomal position and distribution, transcript length, GC content, and exon count, were analyzed and graphically represented using TBtools version 0.665 [60] and GraphPad Prism version 6.01.

### 4.6. Differential Expression Analysis

Cuffdiff was employed to ascertain the differential expression of lncRNA and PCG transcripts. Transcripts were designated as significantly differentially expressed lncRNAs (DElncRNAs) and PCGs (DEPCGs) if they satisfied the thresholds of |log2(fold change)| values ≥ 1, *p* values < 0.05, and q values < 0.05. The Venn diagram, illustrating the overlap and uniqueness of DElncRNAs and DEPCGs, was constructed utilizing the online tool Venny 2.1.0, accessible at https://bioinfogp.cnb.csic.es/tools/venny/index.html (accessed on 6 July 2021). Quantitative and expression level comparisons of the differentially expressed transcripts were conducted using GraphPad Prism version 6.01. For the quantitative comparison, a two-sided Fisher’s exact test was applied, whereas the two-sided Mann-Whitney U test was employed for assessing expression levels. Significance levels were denoted as follows: * *p* < 0.05, ** *p* < 0.01, *** *p* < 0.001, **** *p* < 0.0001.

### 4.7. Prediction of Trans-Regulated Target PCGs of lncRNAs

LncRNAs typically regulate target PCGs through two principal mechanisms: *trans*-regulation and *cis*-regulation. *Trans*-regulation denotes the direct interaction of lncRNAs with PCGs, thereby influencing their functional repertoire. In this study, we screened for calcium-regulated lncRNAs and PCGs as previously described [21]. To construct a weighted gene co-expression network for the selected calcium-regulated lncRNAs and PCGs, we employed the WGCNA shiny plugin of TBtools, which is accessible at the GitHub repository (https://github.com/ShawnWx2019/WGCNA-shinyApp, accessed on 18 January 2023). After importing the gene expression data into the WGCNA shiny plugin, we initiated pre-processing and parameter setting. In the initial filter, we set the sample percentage threshold to 0.9 and established an expression cutoff at 1, thereby excluding genes with FPKM values below 1 across all samples. Subsequent filtering involved the use of Median Absolute Deviation (MAD) as the method, retaining 10,000 gene numbers to mitigate computational intensity. Post data pre-processing, we proceeded with the following parameter settings to derive clustering modules: an R2 cutoff of 0.85, a soft threshold of 30, a minimum module size of 30, a module cut tree height of 0.25, a maximum block size of 10,000, and a cutoff of absolute value of kME at 0.9. Upon identifying modules of interest for further investigation, we conducted Gene Ontology (GO) functional enrichment analysis on the PCGs enriched within these modules to elucidate the biological functions in which the module is implicated. Finally, we employed Cytoscape version 3.7.2 to graphically represent the co-expression network of lncRNAs-PCGs derived from our analysis, providing a visual synthesis of the regulatory relationships uncovered.

### 4.8. Prediction of Cis-Regulated Target PCGs of lncRNAs

*Cis*-regulation, which is contingent upon the genomic proximity between lncRNAs and PCGs, represents an additional layer of gene expression modulation. To identify potential *cis*-regulated target PCGs, we employed Bedtools version 2.31.1, a versatile toolset for genomic feature analysis (https://github.com/arq5x/bedtools2/releases/download/v2.31.1/bedtools-2.31.1.tar.gz, accessed on 7 November 2023). This tool was utilized to select PCGs located within a 100 kb window upstream and downstream of the lncRNAs. Following this, we determined the Pearson correlation coefficient for the expression profiles of lncRNAs and PCGs, screening for lncRNA-PCG pairs with an absolute PCC value of at least 0.9 and a *p*-value less than 0.05, which are considered potential *cis*-regulated target PCGs of lncRNAs. For the visualization of expression patterns, we leveraged the Heatmap Illustrator plugin within TBtools to generate heatmaps using FPKM values. Given the inherently low expression levels of lncRNAs, which can obscure differences when plotted alongside PCGs, we chose to represent the relative expression values of lncRNAs or PCGs in comparison to a zero-expression baseline, focusing on trend visualization. Detailed specific values are provided in Tables S8 and S9. This approach allows for a clearer depiction of the expression trends, while the numerical data complement the graphical representation, offering a comprehensive view of the *cis*-regulatory relationships within the genomic context.

## 5. Conclusions

In the present study, we delved into the role of lncRNAs in *Arabidopsis thaliana* under conditions of salt and hyperosmotic stress imposed with sorbitol, with a particular focus on the impact of cytosolic and nucleosolic calcium signals on lncRNAs and PCGs. Through RNA sequencing, we identified 688 novel lncRNAs and 2223 known lncRNAs that exhibited responsiveness to stress treatments. These findings offer novel insights into the complex regulatory networks underlying plant stress responses. Our analyses revealed distinct regulatory patterns for differentially expressed lncRNAs (DElncRNAs) and protein-coding genes (DEPCGs) in response to salt stress and hyperosmotic stress, with different regional calcium signals exerting inhibitory roles, thereby highlighting the importance of calcium signaling in modulating stress-responsive gene regulation. Co-expression network analysis was instrumental in identifying lncRNA and PCG modules associated with calcium signal responses to stress, thereby shedding light on the intricate regulatory networks integral to plant stress responses. Furthermore, we explored the potential cis-regulation of target PCGs by calcium-regulated lncRNAs, uncovering several lncRNA-PCG pairs that may be involved in cell wall remodeling and the regulation of root morphology in response to osmotic stress. Collectively, this research highlights the significance of lncRNAs in plant stress tolerance and elucidates the complex interplay between calcium signaling and gene regulation in *Arabidopsis thaliana*. This study not only enhances our understanding of the regulatory mechanisms underlying plant stress responses but also provides valuable resources for future research aimed at improving plant stress tolerance.

## Data Availability

The original contributions presented in the study are included in the article/Supplementary Materials, and further inquiries can be directed to the corresponding author/s.

## Supplementary Materials

The following supporting information can be downloaded at: https://www.mdpi.com/article/10.3390/ijms26052086/s1.
