# Supplementary figures and images for: Cytosolic and Nucleosolic Calcium-Regulated Long Non-Coding RNAs and Their Target Protein-Coding Genes in Response to Hyperosmolarity and Salt Stresses in Arabidopsis thaliana
Source: Int J Mol Sci. 2025 Feb 27;26(5):2086. doi: 10.3390/ijms26052086 (PMC11900983; doi:10.3390/ijms26052086)

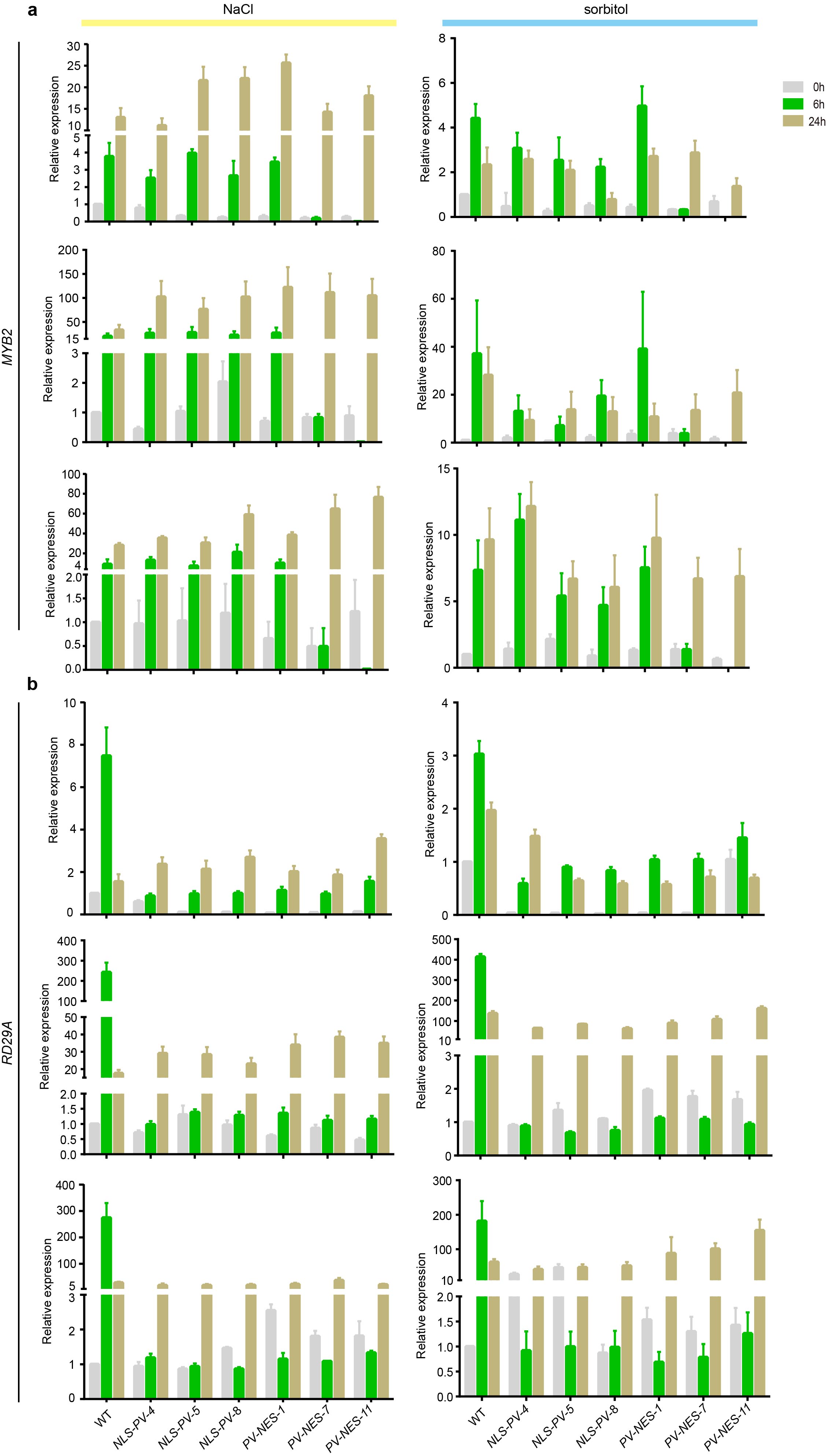

Supplement: Supplementary file 1 [file ijms-26-02086-s001.zip › Supplementary Tables and Figure/FS1.jpg]
